# Exposure to air pollution near a steel plant is associated with reduced heart rate variability: a randomised crossover study

**DOI:** 10.1186/s12940-016-0206-0

**Published:** 2017-01-28

**Authors:** Robin H. Shutt, Lisa Marie Kauri, Scott Weichenthal, Premkumari Kumarathasan, Renaud Vincent, Errol M. Thomson, Ling Liu, Mamun Mahmud, Sabit Cakmak, Robert Dales

**Affiliations:** 1 Population Studies Division, Environmental Health Science Research Bureaum Health Canada, 50 Colombine Driveway, Ottawa, ON K1A 0 K9 Canada; 2 Air Health Sciences Division, Water and Air Quality Bureau, Health Canada, 269 Laurier Ave W, Ottawa, ON K1A 0 K9 Canada; 3 Mechanistic Studies Division, Environmental Health Science Research Bureau, Health Canada, 50 Colombine Driveway, Ottawa, ON K1A 0 K9 Canada; 4 Hazard Identification Division, Environmental Health Science Research Bureau, Health Canada, 50 Colombine Driveway, Ottawa, ON K1A 0 K9 Canada

**Keywords:** Air pollution, Steel production, Heart rate variability, Industrial air pollution, Environment, Epidemiology

## Abstract

**Background:**

Epidemiological studies have shown that as ambient air pollution (AP) increases the risk of cardiovascular mortality also increases. The mechanisms of this effect may be linked to alterations in autonomic nervous system function. We wished to examine the effects of industrial AP on heart rate variability (HRV), a measure of subtle changes in heart rate and rhythm representing autonomic input to the heart.

**Methods:**

Sixty healthy adults were randomized to spend five consecutive 8-h days outdoors in one of two locations: (1) adjacent to a steel plant in the Bayview neighbourhood in Sault Ste Marie Ontario or (2) at a College campus, several kilometers from the plant. Following a 9–16 day washout period, participants spent five consecutive days at the other site. Ambient AP levels and ambulatory electrocardiogram recordings were collected daily. HRV analysis was undertaken on a segment of the ambulatory ECG recording during a 15 min rest period, near the end of the 8-h on-site day. Standard HRV parameters from both time and frequency domains were measured. Ambient AP was measured with fixed site monitors at both sites. Statistical analysis was completed using mixed-effects models.

**Results:**

Compared to the College site, HRV was statistically significantly reduced at the Bayview site by 13% (95%CI 3.6,19.2) for the standard deviation of normal to normal, 8% (95%CI 0.1, 4.9) for the percent normal to normal intervals differing by more than 50 ms, and 15% (95%CI 74.9, 571.2) for low frequency power. Levels of carbon monoxide, sulphur dioxide, nitrogen dioxide, and fine and ultrafine particulates were slightly, but statistically significantly, elevated at Bayview when compared to College. Interquartile range changes in individual air pollutants were significantly associated with reductions in HRV measured on the same day. The patterns of effect showed a high degree of consistency, with nearly all pollutants significantly inversely associated with at least one measure of HRV.

**Conclusions:**

The significant associations between AP and changes in HRV suggest that ambient AP near a steel plant may impact autonomic nervous system control of the heart.

**Electronic supplementary material:**

The online version of this article (doi:10.1186/s12940-016-0206-0) contains supplementary material, which is available to authorized users.

## Background

Elevated exposure to ambient outdoor air pollution (AP) has been shown to contribute to acute and chronic health effects in the Canadian population [1, 2], and globally as well [3–6]. Specifically, chronic exposure to elevated ambient AP has been linked to increased risk of all-cause mortality [6], as well as cardiovascular and pulmonary morbidity and mortality [3, 4]. More recent studies have linked ambient AP to acute effects on human health [2, 4, 7], and more specifically cardiovascular morbidity and mortality, including increased risks of cardiac rhythm disturbance [2]. Data suggest that both acute and chronic effects of AP are related to oxidative stress and activation of stress pathways, including the autonomic nervous system [4].

Heart rate (HR), and variations in HR at the beat-to-beat level are dependent on the activity of the intrinsic cardiac pacemaker, the sino-atrial node [8]. Sino-atrial nodal function and discharge rate are influenced by autonomic inputs, which can be subdivided into the excitatory sympathetic and inhibitory parasympathetic nervous system effects. One approach to look at the balance of sympathetic and parasympathetic function is the study of Heart Rate Variability (HRV) [8, 9]. HRV is determined by autonomic nervous system and circulating hormonal inputs at the sino-atrial node [4, 9, 10]; and is measured by comparing the variations in time between R-waves (ventricular depolarization) from a continuous electrocardiogram [9, 10]. Only normal (sino-atrial nodal) beats are counted, therefore the time between beats is referred to as the normal to normal, or N-N, interval [9, 10]. Reduced HRV has been linked to increased risk of cardiovascular mortality and morbidity in vulnerable populations, including the elderly [11], diabetics and heart failure patients [12], and has been shown to be predictive of all-cause mortality risk in healthy populations [12].

Exposure to single air pollutants, pollution mixtures and/or concentrated particulate matter has been shown to influence HRV. In controlled exposure studies HRV has been shown to be responsive to individual pollutants and pollutant mixtures; specifically ultrafine particulate matter (UFP) [13], concentrated ambient particulate matter, and ozone [14]. In panel and crossover studies of the effects of ambient air pollution on health, significant changes in HRV also have been reported. Significant decreases in high frequency (HF) power of HRV were seen when cyclists cycled in high traffic areas, compared to when they cycled in low traffic or indoors [15]. Nyhan et al. [16] found that particulate matter dose (concentration*respiration rate) was significantly inversely associated with the time domain of HRV in a group of commuters using bus, train, bike or foot transport [16]. Many studies of acute effects of AP exposure on autonomic function through HRV analyses have focused on exposures to traffic-related ambient AP [14–17]; however, few studies have examined the acute effects of exposure to ambient AP from industrial sources.

In the summer of 2010 we conducted a novel ambient AP exposure crossover study. The study compared physiological and biological responses in healthy volunteers who had spent time outdoors either at a location in a neighbourhood, called Bayview, adjacent to a steel mill, or at a college campus several kilometers from the steel mill. Previous reports of the results from this study have focussed on pulmonary function [18] and cardiovascular function [19], as well as the role of metals composition in the effects of particulate matter on pulmonary and cardiovascular function [20]. The current report focuses on associations between proximity to a steel plant and acute (same day) changes in HRV. We hypothesised that (1) HRV would be significantly different at the Bayview site when compared with the College site; (2) AP exposure between the two sites would differ significantly; and (3) that changes in levels of single pollutants would be associated with changes in HRV.

## Methods

### Study design and AP exposure assessment

The study was approved by the Health Canada Research Ethics Board (reference number 2009-0044) and the ethics board at Algoma University. Study design and detailed methods of the study protocol have been reported previously [18–20]. Briefly, eligible study participants were recruited through postings at local university and college campuses. The participants were non-smokers aged 18–55 years, permanent or seasonal residents of Sault Ste. Marie Ontario, who did not work or live near the steel plant. All participants completed an ethics board approved informed consent process. Participants had no history of cardiovascular, respiratory or metabolic disorders and did not use medications that could influence inflammatory processes or cardio-respiratory function. Participants were randomized into two different crossover arms for the study. Participants spent five consecutive eight hour days outdoors at one of two study sites; at the Bayview neighborhood near the steel plant property or at a college campus approximately five km from the plant site. Participants had a minimum of nine days of washout away from the study sites before being assigned to the other exposure scenario. Ambient hourly pollutant levels (CO, NO, NO_2_, NO_x_, O_3_, SO_2_ & PM_2.5_) temperature, humidity and rainfall were determined at each study site using fixed site monitors (Air Pointer, recordum Messtechnik GmbH, Modling, Austria). Ultrafine particles (UFP) were measured using the TSI Model 3007 UFP counter. Daily AP exposures were assigned to study participants based on the mean levels of each pollutant during the time the participant was on site, up to and including the time point at which HRV parameters were measured.

### Measurement of HRV parameters

For measurement of HRV, participants were hooked up to a 12-lead ambulatory ECG monitor (Mortara H12+ Holter Monitor, Mortara Instrument Inc. Milwaukee, WI, USA) by trained technicians following standard procedures [21]. The H12+ system was selected for the high sampling frequency (10 000 samples/sec/channel), as is recommended for research purposes. Data were screened using the Mortara H-scribe workstation, and then transferred to Nevrokard aHRV software (Nevrokard Kiauta, d.o.o., Slovenia) for additional analysis. Data screening was performed by trained ECG technicians to confirm automatically identified ectopic beats, identify and exclude areas of low recording quality on some channels, and to select five minute periods for HRV analysis. Wherever possible the five minute analysis periods were selected at the end of a minimum 15 min sedentary rest period. For the purposes of this paper all data presented are from a 25 min rest period near the end (sixth hour) of the eight hour on-site day. HRV analysis of the listed parameters was completed using the Mortara and Nevrokard software, and following the HRV Measurement Standards established in the 1996 European Society of Cardiology and The North American Society of Pacing and Electrophysiology guidelines [9, 10]:

- Time Domain: SDNN - Standard Deviation of the N to N intervals, in ms.
  RMSSD - Root mean square of the sucessive differences in ms.
  pNN50 - The percentage of pairs of adjacent N to N intervals that differ by more than 50 ms.

- Frequency Domain: Analysis of the frequency domain was completed using non-parametric, Fast Fourier transformation methods.
  LF Power – Power in the low frequency range (0.04–0.15 Hz), in ms^2^.
  HF Power – Power in the high frequency range (0.15–0.4 Hz), in ms^2^.

Additionally, heart rate (Normal beats per minute) was calculated for the HRV analysis period for use as a covariate in HRV analysis, and the ratio of LF power to HF power (LF/HF) was calculated for inclusion in the Frequency Domain data analysis [9, 10].

### Statistical analyses

Statistical analysis methods were similar to those described in Dales et al. [18], however only acute (same day) associations were considered.

#### Analysis of site and HRV

Associations between study site and HRV parameters were measured with mixed effects linear regression models, matched for study participant and day of the week. Covariates in the analyses included HR, age, sex, body mass index, temperature and relative humidity. Demographic, HRV and exposure databases were merged and statistical analyses were completed with SAS EG, v4.2 (Cary, NC, USA).

#### Analysis of site differences in AP levels

The levels of exposure to pollution for participants at the Bayview site were compared to their exposure levels at the College site with student’s t-tests.

#### Analysis of individual pollutants and HRV

Associations between individual daily average pollutant exposure concentrations and daily changes in HRV for the two sites combined were determined. Individual daily pollutant exposures were determined based on the mean exposure levels for the specific participant’s time at the study site up to and including the hour of the HRV time point. The associations between concentrations of individual pollutants and HRV parameters measured on the same day were examined using mixed effects linear regression models. Covariates in this analysis included HR, age, sex, BMI, temperature and relative humidity with the addition of study site. Sample size was inadequate to complete subgroup analyses. The final model contained the selected variables and covariates if they were significant at *p* <0.05 or if they confounded the exposure-outcome relationship (i.e. a change of 10% in the coefficient for exposure).

## Results

**Fig. 1:**
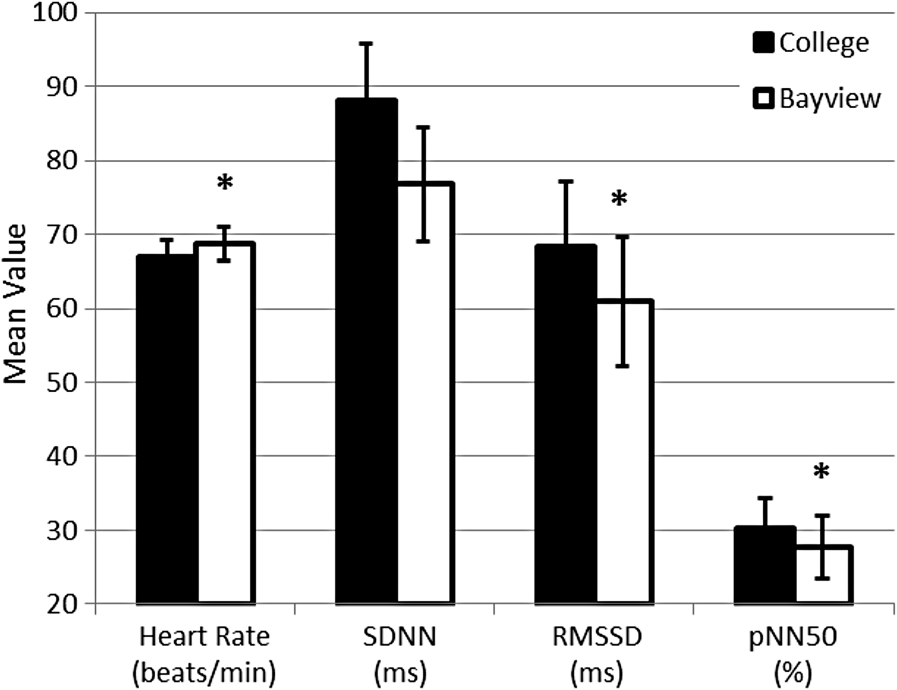
Heart Rate and Time Domain of Heart Rate Variability differed significantly at the Plant site when compared to the College site. Data are expressed as the mean of responses measured from the afternoon ambulatory ECG recording time point. Error bars represent the 5 and 95% confidence limits. Mixed effects linear regressions showed that the increase in heart rate at the Plant Site, when compared to the College Site was significant (* = *p* < 0.05). The decreases in SDNN and pNN50 at the Plant Site, when compared to the College Site also were significant

**Fig. 2:**
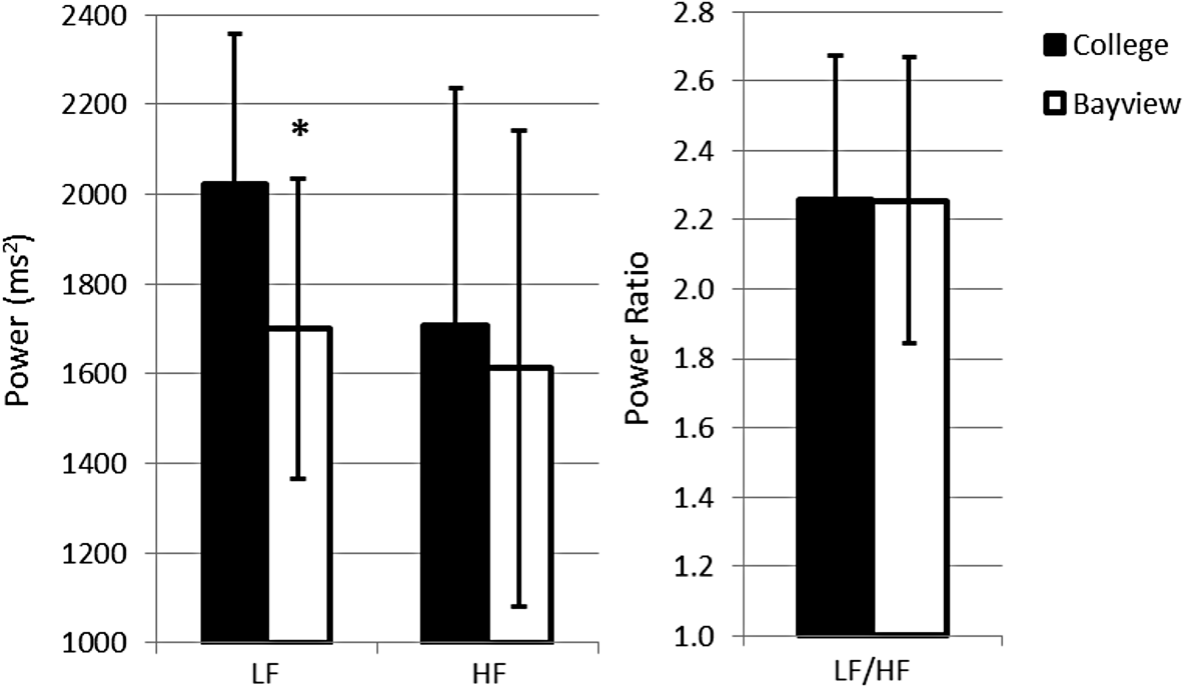
Low Frequency Power was significantly reduced at the Plant site. Data are expressed as the mean of responses measured from the afternoon ambulatory ECG recording time point. Error bars represent the 5 and 95% confidence limits. Mixed effects linear regression determined that LF power was significantly reduced (* = *p* < 0.05) at the college site when compared to the plant site, while HF power and the LF/HF ratio did not differ significantly

Sixty participants completed at least part of each of the 5-day exposure scenarios (Bayview and College sites) and were included in the statistical analyses presented here. Descriptive statistics for the cohort are presented in Table 1. We compared afternoon HR and HRV parameters at the Bayview site with HR and HRV at the College site to test our first hypothesis, that cardiac autonomic balance, as described by HRV, would be altered at the Bayview site. HR was slightly (2.8%) but significantly elevated at the Bayview site, when compared to the College site (Fig. 1). Components of the time domain of HRV decreased significantly at Bayview when compared to College (Fig. 1). SDNN and pNN50 were significantly reduced at the Bayview site when compared to the College site. SDNN was reduced by nearly 13%, while pNN50 showed an 8.2% reduction (Mean and confidence intervals are reported in Fig. 1). The frequency domain of HRV also was affected by site (Fig. 2). There was a significant reduction (8.4%) in LF power at the Bayview site (Fig. 2) when compared to the College site; however there was no effect of site on either the HF power or the LF/HF ratio.

**Table 1:** Participant demographics

	Mean	SD
Age (years)	24.2	5.8
Height (cm)	173.1	9.9
Weight (kg)	78.7	20.2
BMI	26.1	5.8
	Percentage	N
Sex, Male	46.0	28

More detailed descriptions of the participant population can be found in Dales et al., 2013. Age in years, height in centimetres, weight in kilograms and Body Mass Index (BMI) are presented as the mean for the 60 participants included in the analysis, with the standard deviation in the second column. 28 participants (46%) were male

Our second hypothesis was that, given the proximity of the Bayview site to the steel plant, AP exposure would differ significantly between the Bayview and College sites. In order to examine this hypothesis we first looked at the participant specific ambient AP exposures, by site (Table 2). AP exposure differed significantly between sites; mean levels of CO, NO, NO_2_, NO_x_, SO_2_, PM_2.5_ and UFP all were significantly elevated at the Bayview site, adjacent to the steel plant. Ozone was significantly lower at the Bayview site, but only represented an 8% difference in O_3_ levels when compared to the College site.

**Table 2:** Comparison of ambient AP levels measured at the college and Bayview Sites

	College site		Bayview site	
Pollutant	Mean (SD)	IQR	Mean(SD)	IQR
CO (ppm)	0.42 (0.11)	0.20	1.24 (1.63)*	0.60
NO (ppb)	1.69 (1.2)	1.30	7.02 (4.16)*	4.90
NO_2_ (ppb)	4.6 (2.8)	2.90	6.89 (3.64)*	4.60
NO_x_ (ppb)	6.29 (3.66)	2.95	13.74 (6.97)*	8.70
O_3_ (ppb)	32.44 (7.62)	8.90	29.66 (6.53)*	9.90
SO_2_ (ppb)	1.78 (2.56)	1.60	8.05 (10.58)*	14.20
PM_2.5_ (μg/m^3^)	11.67 (6.61)	8.20	13.01 (6.87)*	9.90
UFP (particles/cm^3^)	6960 (4261)	5560	14654 (13664)*	19648

Mean values are the mean for the participant specific exposure averaged over the hours of the five days that each individual participant spent breathing ambient air at the specified site. Pollutant levels were significantly different between the College and Bayview sites (* = *p* < 0.05)

Mean temperature at College 23.4 °C (SD 4.1 °C); mean relative humidity 56.0% (SD 15.1%)

Mean temperature at Bayview 23.4 °C (SD 4.1 °C); mean relative humidity 55.9% (SD 14.3%)

Once it was determined that the AP exposures at the two sites differed, we tested our third hypothesis; that changes in levels of individual pollutants would be associated with changes in HRV, regardless of site. For this analysis participant specific AP exposures were determined to investigate the effects of interquartile range (IQR) changes in individual pollutant exposures on HRV (Table 3 shows IQR for each pollutant, exposures at both sites were combined). The models predicted small but significant increases in HR for IQR (see Table 3) changes in CO, NO, NO_2_, NO_x_, SO_2_ and UFP. The time domain of HRV, including SDNN, RMSSD and pNN50, was significantly associated with IQR increases in pollutant levels (Table 3). Associations between the time domain of HRV and IQR changes in pollutant levels were consistently inverse (increasing levels of pollutants associated with decreasing HRV). IQR changes in all pollutants but ozone were significantly associated with small (1–10%) but significant reductions in SDNN. IQR changes in NO, PM_2.5_ and UFP were associated with consistent (~5%) reductions in pNN50. Significant inverse associations between individual pollutant levels and RMSSD were seen with NO_2_, NO_x_, SO_2_ and PM_2.5_ (Table 3). No significant positive associations between any of the reported pollutants and the Time Domain of HRV were found (Table 3).

**Table 3:** Associations between IQR increases in levels of individual pollutants and components of heart rate variability

			Frequency domain			Time domain		
Pollutant	Pollutant IQR	Heart Rate (bpm)	HF Power (ms^2^)	LF Power (ms^2^)	HF/LF	SDNN (ms)	RMSSD (ms)	pNN50 (%)
CO (ppm)	0.30	0.32* (0.10, 0.54)	-0.39 (-0.89, 0.11)	-3.78* (-7.41, -0.12)	-0.03 (-0.08, 0.02)	-1.09* (-2.12, -0.06)	-0.99 (-2.09, 0.11)	-0.38 (-0.80, 0.03)
NO (ppb)	4.40	1.22* (0.31, 2.14)	-0.56 (-2.61, 1.50)	-1.51* (-3.01, -0.01)	-0.09 (-0.31, 0.13)	-4.67* (-8.89, -0.44)	-4.13 (-8.62, 0.35)	-1.77* (-3.52, -0.02)
NO_2_ (ppb)	4.30	1.40* (0.48, 2.32)	-1.101 (-3.17, 0.97)	-1.33 (-2.82, 0.15)	-0.19 (-0.40, 0.03)	-6.41* (-10.65, -2.17)	-6.02* (-10.53, -1.52)	-1.17 (-2.94, 0.59)
NO_x_ (ppb)	8.30	1.50* (0.56, 2.43)	-0.38 (-2.46, 1.71)	-1.46 (-3.00, 0.08)	-0.12 (-0.34, 0.09)	-6.60* (-10.90, -2.29)	-6.01* (-10.58, -1.44)	-1.41 (-3.21, 0.38)
O_3_ (ppb)	8.70	0.30 (-0.66, 1.26)	-2.50* (-4.67, -0.33)	-2.24 (-17.32, 12.84)	-0.32* (-0.54, -0.11)	-5.59 (-10.01, 1.18)	-6.11 (-10.87, 1.36)	1.28 (-0.55, 3.10)
SO_2_ (ppb)	4.60	0.78* (0.15, 1.41)	-0.82 (-2.24, 0.61)	-1.07* (-2.13, -0.002)	-0.11 (-0.25, 0.03)	-4.08* (-7.00, -1.16)	-3.41 (-6.52, -0.31)*	-1.08 (-2.30, 0.13)
PM_2.5_ (μg/m^3^)	9.00	0.97 (-0.04, 1.97)	-2.43* (-4.62, -0.24)	-1.88* (-3.46, -0.29)	-0.16 (-0.38, 0.07)	-5.34 * (-9.96, -0.72)	-5.28* (-10.15, -0.41)	-1.99* (-3.91, -0.07)
UFP (particles/cm^3^)	12236	1.10* (0.04, 2.16)	-1.89 (-4.38, 0.60)	-1.61* (-3.21, -0.01)	-0.15 (-0.38, 0.08)	-7.13* (-12.27, -1.98)	-5.03 (-10.63, 0.57)	-2.20* (-4.24, -0.15)

IQR values for participant specific pollutant exposures, combined for both the Bayview and College sites, are shown in the second column. The amplitude of the change in each HRV parameter associated with an IQR increase in pollutant level and 95% CI (bracketed) are reported for each pollutant/HRV parameter pair. Statistically significant associations (*p* < 0.05) are denoted by *

* = *p* < 0.05. Mean temperature throughout the study was 23.4 °C (SD 4.1 °C); mean relative humidity was 56.0% (SD 14.7%)

We also saw significant associations between changes in levels of individual pollutants and the Frequency Domain of HRV (Table 3). IQR increases in CO, NO, SO_2_, PM_2.5_ and UFP all were significantly inversely associated with LF power. It should be noted that these associations, while statistically significant, represent small changes in LF Power (0.1–0.2% reductions) when compared with those associated with the College vs Bayview sites. The patterns of associations, while generally inverse, between IQR range changes in AP and HF power and the ratio of HF to LF power were less clearly defined, and few reached statistical significance. Small but significant inverse associations were found between HF power and O_3_ and PM_2.5_, as well as a significant inverse association between O_3_ and HF/LF. No significant positive associations or trends towards positive associations were seen in the Frequency Domain of HRV (Table 3).

We also tested to determine if individual pollutant levels were correlated with one another. We pooled the pollutant exposure data, irrespective of site, and examined the Pearson Product Moment Correlations between pollutants (Additional file 1: Table S1). Levels of both NO and NO_2_ were very strongly correlated with NO_X_ levels. NO and NO_2_ were strongly correlated. NO_2_ and NO_X_ also showed strong correlations with levels of SO_2_. Levels of NO_2_ were moderately correlated with levels of particulates (both fine and ultrafine). NO_X_ levels were moderately correlated with the concentration of ultrafine particles. SO_2_ was strongly correlated with levels of both NO_2_ and NO_x_, and was weakly correlated with NO. SO_2_ also was very strongly correlated with concentrations of UFP and moderately correlated with fine particulate concentrations. O_3_ was moderately inversely correlated with NO. Otherwise, only weak correlations between levels of O_3_ the other pollutants measured were seen, and most of those correlations were inverse correlations, rather than the positive correlations between the other pollutants (Additional file 1: Table S1).

## Discussion

In this study we investigated the effects of acute exposure to ambient AP from an industrial source on cardiac autonomic function, as described by changes in HRV. We found small but statistically significant associations between exposure to ambient AP at a site near a steel plant and reductions in both time and frequency domain components of HRV. The associations between exposure sites and HRV appear to be related to the statistically significant pollutant exposure contrast between the two sites, as similar associations were seen in models of the effects of individual pollutant exposures on HRV. Associations between increasing levels of AP and the time domain showed a consistent inverse relationship, HRV decreasing as pollutant levels increased. A similar pattern of associations between AP and the frequency domain was seen although this may be more challenging to interpret.

An initial report of the effects of ambient AP near the steel plant on cardiovascular outcomes showed that pulse rate (PR) was slightly but significantly elevated (approximately 1.5 bpm) at the plant site [19]. Liu et al. [19] described significant elevations in PR in response to IQR increases in SO_2_ (IQR = 2.9 ppb), NO_2_ (IQR = 5.0 ppb) and CO (IQR = 0.2 ppb). In the current investigation of the effects of ambient AP near the steel plant on autonomic function, we also showed a small but significant elevation in HR at the Bayview site, when compared to the College site (approximately 2 bpm; Fig. 1) and small but consistently significant associations between individual pollutants and HR (Table 3). While we saw no association between IQR changes in O_3_ and HR, Liu et al. [19] did report a significant inverse association. The difference in this finding, as well as the additional finding of significant associations between HR and IQR changes in NO, NO_x_ and UFP concentrations in this study may be related to the difference in the sensitivity and recording duration for HR. In the Liu et al. [19] report, PR was derived from the PR recorded during pulse oximetry at a different time point than the HR in the current analysis, which was determined from an ambulatory electrocardiogram. In addition, participant specific AP exposure estimates were based on slightly different exposure times than those used by Liu and colleagues [19]. Finally, we might expect some small differences in results due to the subtle differences in the mixed effects models used for data analysis, and the number of participant records available for analysis for each variable and at each time point. In our analysis IQR increases of most of the measured pollutants (excluding O_3_) were significantly positively associated with HR and significantly negatively associated with the time domain of HRV. Increased HR and decreased HRV are hallmarks of increased sympathetic input [22].

The effects of exposure to ambient AP from industrial sites on HRV in healthy young populations have not been extensively studied, and the effects seen in other studies of AP mixtures or single pollutants show a high degree of heterogeneity, with few points of consensus. For example, in two similar crossover studies of the effects of traffic related AP exposure on HRV parameters, Weichenthal et al. [15, 17] found different patterns of effects. In a 2011 study of urban cyclists Weichenthal et al. [15] found that HF power was significantly reduced when cycling in high traffic areas, and HRV was inversely associated with IQR increases in UFP concentrations [15]. The reported reductions in HRV in response to increasing pollutant levels in Weichenthal et al. [15] support the findings of the current study. In 2014 Weichenthal et al. [17] reported that HRV was affected by traffic related AP in a population of female cyclists, but the results were unexpected in the context of the 2011 study [15, 17]. Specifically, ozone was associated with significant increases in SDNN and LF/HF ratio, and more surprisingly UFPs were associated with a significant increase in SDNN [17]. Additionally, regional background AP levels appeared to be modifying autonomic nervous system sensitivity to short term traffic pollution exposures, suggesting that an individual’s background AP exposures may modulate acute responses to traffic-related AP [17]. The consistent inverse associations we report in this study are not supported by the findings of the Weichenthal et al. 2014 [17] study. In another study of healthy young commuters, Nyhan et al. [16] found that exposure to particulate matter was associated with significant reductions in the time domain of HRV. This finding was stronger in physically active commuters (cyclists and pedestrians) but also was true for more sedentary commuters (bus and train) [16]. These findings support the findings of the current study, which showed significant reduction in the time domain of HRV associated with IQR increases in PM_2.5_.

Other studies have investigated the effects of controlled exposures to components of AP on HRV in healthy subjects. In controlled exposures of healthy young volunteers to carbon UFP, significant positive associations between UFP and HRV parameters were reported [13]. The pattern of effects suggested that peripheral nervous system tone was elevated following exposure to low levels of UFP, but not at higher concentrations [13]. In our study, when the associations between HRV parameters and IQR changes in UFP were tested, we saw significant inverse associations with some measures, and all trends were consistently inverse, suggesting an increase in sympathetic tone. Controlled exposure to concentrated ambient particles [14] was positively associated with the frequency domain of HRV, and these changes were larger following co-exposure to particles and O_3_. Individual pollutants may interact in ambient pollutant mixtures to enhance or alter the autonomic effects of AP.

In future analyses it may be important to consider interactions between pollutants or to generate multi-pollutant models to better test the associations between AP and autonomic effects. We tested the degree of correlation between individual pollutants irrespective of site, and found that most pollutants were significantly positively correlated with one another. We found consistent, moderate to strong, positive correlations between the Nitrogen oxides and also SO_2_ (Additional file 1: Table S1). This, taken with our findings of consistent inverse associations between these individual pollutants and HRV, suggests that the high degree of correlation between individual pollutants may contribute to the consistency of the effect. It may be important to investigate the effects of controlled exposure to individual pollutants and specific mixtures to tease out which pollutants are primarily responsible for the effects on HRV. However, if we consider our site-specific analysis as a method to investigate the effects of pollutant mixtures on HRV, the pollutant mixture at the Bayview site was associated with significant reductions in HRV when compared to the mixture at the College site. We also took a second multipollutant approach, by testing the associations between IQR changes in the Air Quality Health Index (AQHI) and HRV parameters. The AQHI is a health information tool, calculated from ambient O_3_, NO_2_, and PM_2.5_ [23]. It is used to communicate the health risks of AP to the public. Previous studies [2, 24–27], however, also have used the AQHI as a unique 3 pollutant summary of AP, and tested its associations with health effects, including asthma [25, 26] and cardiovascular outcomes [2, 24]. In our study the AQHI was not significantly associated with changes in any of the HRV parameters tested (Additional file 2: Table S2). A detailed description of the AQHI and the results can be found in the table and legend for Additional file 2: Table S2.

Healthy younger individuals have been followed in two different panel studies of ambient AP effects in Taiwan [28, 29]. Chuang et al. [28] reported consistent inverse associations between ambient AP levels and both time and frequency domains of HRV. These results were similar to the results of the current study, although some of the patterns of effect differed. Chuang et al. [28] also reported associations between AP and elevated blood levels of biomarkers of oxidative stress, suggesting that oxidative stress may be involved in the altered autonomic function. In mail carriers in Taiwan, IQR changes in PM_2.5_ were not significantly associated with changes in HRV [29]. The lack of significant associations reported by Wu et al. [29] are especially surprising in light of the much higher exposure experienced by the mail carriers (PM_2.5_ IQR = 52) when compared to the exposures in the current study (IQR = 9), where we reported significant inverse associations. It is possible that the lack of significant associations in the mail carriers study [29] was related to a lack of exposure contrast, since no crossover to a different exposure scenario was completed, and background exposures may have been similar or higher. Taken together, the results of the current study with results from other studies in healthy younger study populations [13–17, 28, 29] appear to show that depending on pollutants, exposure metric, and timeline the effects of AP on autonomic nervous system function can show slightly different patterns of effects; however throughout all the studies, the overall message appears to be that exposure to AP can alter cardiac autonomic balance in the absence of other disease states or risk factors.

When we examined the associations between LF power and site alone or LF power and individual pollutants, LF showed significant inverse associations. The inputs which might influence changes in LF power are less clearly defined than those for HF power or the ratio of LF:HF. Changes in HF power are consistently linked to changes in parasympathetic input [8, 9, 22]. Some researchers therefore have hypothesized that changes in LF power might be evidence of sympathetic effects [22], while others suggest that such change represents both sympathetic and parasympathetic activity [9] but there is no clear consensus, and it is difficult to interpret the individual findings with respect to LF power. Other studies of healthy younger volunteers have reported significant changes in LF power [14, 28], in the absence of effects on other components of the frequency domain of HRV. While reductions in HF were linked to pollutant and particulate matter exposure in diabetics [30], the elderly [31] and individuals with a history of hypertension or ischemic heart disease [31], this was not seen in healthy young volunteers [30]. These findings seem to point to a difference in the response to pollutant exposure of the frequency domain. LF power appears to be inversely associated with increasing AP in healthy young study participants in this study and others [14, 28], while AP exposure in members of vulnerable populations has been associated with changes in HF power [30, 31].

The associations between AP and HRV reported in this study were small. This may be related to the study population, who were a group of healthy and relatively young volunteers, rather than members of susceptible populations. The changes in HRV were statistically significant and followed consistent patterns of effect, especially within the time domain of HRV. The consistent patterns of association suggest that these effects represent real changes in autonomic function in response to changing AP rather than spurious associations. Ambient AP levels in this study were consistent with generally low Canadian ambient AP levels [32], and therefore are relevant to Canadians. Additionally, the outdoor exposure location close to the steel plant was adjacent to a residential neighbourhood; therefore, ambient AP exposures at the Bayview site reflected real world ambient AP exposure. It is possible that, had pollutant levels been higher, and the exposure contrast been greater, a larger effect of proximity to the industrial site, and of pollutant exposure, on autonomic nervous system function could have been recorded. That said, our statistically significant findings at relatively low levels of pollution, and with a small exposure contrast between study sites, provide us with important information about the autonomic effects of small changes in AP when AP levels are low. The crossover design of the study was a great strength, allowing us to contrast the health effects of exposure to AP near a steel plant and at a site farther from the plant. In the crossover design participants act as their own controls, reducing the potential for confounding by time-invariant individual characteristics such as age, sex, social status, residential air quality, and medical history. Some bias may have been introduced into the study due to the nature of the crossover design. It was not possible to blind the study participants or study team to the exposure arm, as the exposure was location specific, and the Bayview site was within view of the steel plant. The analysis of associations with individual pollutant levels, however, which pooled all pollutant exposures by participant irrespective of site, would have mitigated some of the bias introduced by the site dependence of the primary study design. The Sault Ste Marie Crossover study as a whole was a randomized controlled crossover study where the exposure conditions were not artificial, but rather were real world exposures, and therefore the significant results that this study has yielded may be generalizable to the Canadian population.

The results of our study, taken together with other studies, indicate that the patterns of changes in autonomic function associated with exposure to AP may differ between young healthy individuals and vulnerable individuals. Reductions in HF power are linked to pollutant and particulate matter exposure in diabetics [30] and the elderly [31], while this study, as well as Fakhri et al. [14] and Chuang et al. [28] reported few associations between AP and HF power, but significant inverse associations with LF power, all in healthy, younger populations. Despite the less clear pattern of significant associations with the frequency domain of HRV, HR and the time domain of HRV showed consistent significant inverse associations with changes in HRV. This is indicative of either reduced parasympathetic activation or enhanced sympathetic activation [22]. Increased sympathetic activation could be indicative of increased systemic stress, and would have wide ranging physiological consequences. Recent animal studies have shown that hypothalamic-pituitary axis and the autonomic nervous system may be activated by inhaled particulate and/or gaseous AP [33, 34]. Recent studies have pointed for a role of increased sympathetic activity in the health effects of AP [4, 34], however these alterations in autonomic function may be a direct mechanism of the health effects of ambient AP, or they may be a secondary effect of activation of oxidative stress pathways [4, 28].

## Conclusions

In our study, cardiac autonomic function appeared to be altered in both a site dependent and a pollutant dependent manner. Proximity to the steel plant was associated with significant reductions in HRV, and these reductions appeared to be related to the elevated pollutant concentrations at the Bayview site. Reductions in HRV were consistent and statistically significant, and they may provide a window into the mechanisms driving the systemic effects of acute exposure to AP. Increased sympathetic nervous system activity is associated with reductions in HRV, such as those seen in our study, and with increased systemic stress. Whether the autonomic effects of site, or ambient pollution are a direct result of pollutant exposure or whether they are secondary effects of other systemic responses to AP, such as oxidative stress, cannot be determined from this study; however it is clear that exposure to ambient AP is associated with altered autonomic function, and specifically elevated sympathetic tone. Reduced HRV has been linked to increased risk of cardiovascular mortality and morbidity in vulnerable populations and has been shown to be predictive of all-cause mortality risk in healthy populations [11, 12], and therefore may represent a mechanistic link to the AP-mediated changes in risk of mortality and morbidity, specifically cardiovascular mortality and morbidity. Finally, it appears that we have found evidence of a pattern of effects of AP exposure on the frequency domain of HRV in young healthy volunteers, which differs from the pattern of effects reported in studies of vulnerable populations.

## Additional files

Additional file 1: Table S1. Correlations between pollutants measured in the Sault Ste Marie crossover study, irrespective of site. (DOCX 20 kb)
Additional file 2: Table S2. Associations between IQR increases in daily maximum Air Quality Health Index and components of heart rate variability. (DOCX 17 kb)

## Abbreviations

AP: Air pollution
BMI: Body mass index
CO: Carbon monoxide
HF: High frequency
HR: Heart rate
HRV: Heart rate variability
IQR: Interquartile range
LF: Low frequency
N: Normal (sinoatrial node derived) beat
NO: Nitric oxide
NO_2_: Nitrogen dioxide
NO_X_: Nitrogen oxides
O_3_: Ozone
PM_2.5_: Particulate matter with an aerodynamic diameter of 2.5 μm or less
pNN50: Percent of N-N intervals differing by more than 50 ms
PR: Pulse rate
RMSSD: Root mean square of sucessive differences
SDNN: Standard deviation of N-N intervals
SO_2_: Sulphur dioxide
UFP: Ultrafine particulate matter
