# Supplementary material for: Exposure to air pollution near a steel plant is associated with reduced heart rate variability: a randomised crossover study
Source: Environ Health. 2017 Jan 28;16:4. doi: 10.1186/s12940-016-0206-0 (PMC5273798; doi:10.1186/s12940-016-0206-0)
Supplement: Additional file 1: Table S1. — Correlations between pollutants measured in the Sault Ste Marie crossover study, irrespective of site. (DOCX 20 kb) [file 12940_2016_206_MOESM1_ESM.docx]

**Additional file 1: Table S1: Correlations between pollutants measured in the Sault Ste Marie crossover study, irrespective of site.** Pearson Product-Moment Correlation Coefficients were determined for each pollutant pair. For each pollutant pair in the table, the following parameters are reported: Pearson R (correlation coefficient), the probability of the correlation being equal to 0 (the null hypothesis), and N, the number of participant*days of exposure data available for correlation. ***Very Strong*** (0.8-1.0) correlations are presented in bold and italics, **Strong** (0.6-0.79) in bold, and *Moderate* (0.4-0.59) in italics.

|  |  | CO | NO | NO_2_ | NO_X_ | O_3_ | SO_2_ | PM_2.5_ | UFP |
| --- | --- | --- | --- | --- | --- | --- | --- | --- | --- |
| CO | Pearson R |  | 0.094 | 0.071 | 0.091 | 0.088 | 0.315 | 0.310 | 0.253 |
|  | Probability |  | 0.0295 | 0.1002 | 0.0363 | 0.0412 | <.0001 | <.0001 | <.0001 |
|  | N |  | 535 | 535 | 535 | 535 | 535 | 535 | 349 |
| NO | Pearson R | 0.094 |  | **0.607** | ***0.906*** | *-0.442* | *0.532* | 0.062 | 0.347 |
|  | Probability | 0.0295 |  | **<.0001** | ***<.0001*** | *<.0001* | *<.0001* | 0.1531 | <.0001 |
|  | N | 535 |  | **535** | ***535*** | *535* | *535* | 535 | 349 |
| NO_2_ | Pearson R | 0.071 | **0.607** |  | ***0.885*** | -0.127 | **0.673** | *0.433* | *0.512* |
|  | Probability | 0.1002 | **<.0001** |  | ***<.0001*** | 0.0032 | **<.0001** | *<.0001* | *<.0001* |
|  | N | 535 | **535** |  | ***535*** | 535 | **535** | *535* | *349* |
| NO_X_ | Pearson R | 0.091 | ***0.906*** | ***0.885*** |  | -0.33 | **0.671** | 0.264 | *0.483* |
|  | Probability | 0.0363 | ***<.0001*** | ***<.0001*** |  | <.0001 | **<.0001** | <.0001 | *<.0001* |
|  | N | 535 | ***535*** | ***535*** |  | 535 | **535** | 535 | *349* |
| O_3_ | Pearson R | 0.088 | *-0.442* | -0.127 | -0.33 |  | -0.142 | 0.237 | -0.222 |
|  | Probability | 0.0412 | *<.0001* | 0.0032 | <.0001 |  | 0.001 | <0.0001 | <0.0001 |
|  | N | 535 | *535* | 535 | 535 |  | 535 | 535 | 349 |
| SO_2_ | Pearson R | 0.315 | *0.532* | **0.673** | **0.671** | -0.142 |  | *0.437* | ***0.802*** |
|  | Probability | <.0001 | *<.0001* | **<.0001** | **<.0001** | 0.001 |  | *<.0001* | ***<.0001*** |
|  | N | 535 | *535* | **535** | **535** | 535 |  | *535* | ***349*** |
| PM_2.5_ | Pearson R | 0.310 | 0.062 | *0.433* | 0.264 | 0.237 | *0.437* |  | 0.397 |
|  | Probability | <.0001 | 0.1531 | *<.0001* | <.0001 | <0.0001 | *<.0001* |  | <.0001 |
|  | N | 535 | 535 | *535* | 535 | 535 | *535* |  | 349 |
| UFP | Pearson R | 0.253 | 0.347 | *0.512* | *0.483* | -0.222 | ***0.802*** | 0.397 |  |
|  | Probability | <.0001 | <.0001 | *<.0001* | *<.0001* | <0.0001 | ***<.0001*** | <.0001 |  |
|  | N | 349 | 349 | *349* | *349* | 349 | ***349*** | 349 |  |
