# Supplementary material for: Exposure to air pollution near a steel plant is associated with reduced heart rate variability: a randomised crossover study
Source: Environ Health. 2017 Jan 28;16:4. doi: 10.1186/s12940-016-0206-0 (PMC5273798; doi:10.1186/s12940-016-0206-0)
Supplement: Additional file 2: Table S2. — Associations between IQR increases in daily maximum Air Quality Health Index and components of heart rate variability. (DOCX 17 kb) [file 12940_2016_206_MOESM2_ESM.docx]

**Additional file 2: Table S2: Associations between IQR increases in Daily Maximum Air Quality Health Index and components of Heart Rate Variability.** The Air Quality Health Index (AQHI) is an indicator of the short-term health risks associated with air quality that is based on mortality data from large urban centres and concentrations three air pollutants, Ozone, PM2.5 and Nitrogen Dioxide. The AQHI is calculated on a 3-hr trailing average of the pollutant concentrations using the following formula:

AQHI = (100*10/10.4)*(e^(0.000537*[Ozone])^ + e^(0.000487*[PM2.5])^ + e^(0.000871*[Nitrogen Dioxide])^ ‑3).

The AQHI was developed as a health information tool [23], however more recent studies have tested associations between calculated AQHI and morbidity, using AQHI as a surrogate for the individual pollutant exposures [2, 24, 25, 26, 27]. Participant specific mean Daily Maximum calculated AQHI (6-hour participant specific time window) as well as minimum and maximum values (all values are combined for both the Bayview and College sites), are shown in the first column. IQR value for participant specific Daily Maximum AQHI, is shown in the second column. The amplitude of the change in each HRV parameter associated with an IQR increase in pollutant level and 95% CI (bracketed) are reported for each pollutant/HRV parameter pair. No statistically significant associations (p < 0.05) were found.

|  |  |  | Frequency Domain | | | Time Domain | | |
| --- | --- | --- | --- | --- | --- | --- | --- | --- |
| Daily Max. AQHI  (min, max) | AQHI IQR | Heart Rate (bpm)  (95% CI) | HF Power (ms^2^)  (95% CI) | LF Power (ms^2^)  (95% CI) | HF/LF  (95% CI) | SDNN  (ms)  (95% CI) | RMSSD  (ms)  (95% CI) | pNN50  (%)  (95% CI) |
| 2.9  (1.43, 5.19) | 0.87 | 0.58  (-0.84, 1.99) | 40.77 (‑247.05, 328.59) | -101.24 (‑330.17, 127.69) | -0.18  (-0.50, 0.13) | -1.48  (-8.52, 5.55) | -0.74  (-8.14, 6.65) | 0.22  (-2.34, 2.85) |

AQHI = (100*10/10.4)*(e^(0.000537*[Ozone])^ + e^(0.000487*[PM2.5])^ + e^(0.000871*[Nitrogen Dioxide])^ ‑3)

No significant (p < 0.05) associations between Daily Maximum AQHI and changes in HRV.

Mean temperature throughout the study was 23.4^o^C (SD 4.1 ^o^C); mean relative humidity was 56.0% (SD 14.7%).
